# Factors associated with cerebrospinal fluid abnormalities suggestive of neurosyphilis in HIV-negative patients with secondary syphilis: a retrospective study

**DOI:** 10.3389/fmed.2026.1839066

**Published:** 2026-05-04

**Authors:** Lin Zhu, Xin Gu, Liyan Ni, Wei Zhao, Zhifang Guan, Haikong Lu, Pingyu Zhou

**Affiliations:** Department of Sexually Transmitted Disease Institute, Shanghai Skin Disease Hospital, Tongji University School of Medicine, Shanghai, China

**Keywords:** CD4 T lymphocytes, cerebrospinal fluid, neurosyphilis, oral mucosal lesions, risk factors, secondary syphilis

## Abstract

**Objective:**

To identify clinical and laboratory factors associated with cerebrospinal fluid (CSF) abnormalities suggestive of neurosyphilis (NS) among HIV-negative patients with secondary syphilis.

**Methods:**

We conducted a retrospective analysis of 805 HIV-negative patients diagnosed with secondary syphilis who underwent lumbar puncture (Lp) at a tertiary referral center in Shanghai, China, between 2009 and 2022. NS was defined based on CSF-VDRL reactivity and/or CSF abnormalities (elevated protein or pleocytosis) in the absence of alternative causes. Logistic regression models were used to identify factors associated with NS.

**Results:**

Among 805 participants, 402 (49.9%) met the study definition of NS. In multivariable analysis, age > 39 years [adjusted OR (aOR) = 2.72, 95% CI 2.01–3.68], male patients (aOR = 2.14, 95% CI 1.58–2.90), presence of oral mucosal patches (aOR = 2.21, 95% CI 1.38–3.55), and CD4^+^T cell counts < 500 cells/μL (aOR = 1.46, 95% CI 1.02–2.10) were independently associated with NS.

**Conclusion:**

In HIV-negative patients with secondary syphilis undergoing Lp, several demographic, clinical, and immunological factors were associated with CSF abnormalities suggestive of NS. These findings may assist clinicians in identifying patients who could benefit from further neurological evaluation. However, given the broad case definition and potential selection bias, the results should be interpreted with caution. Prospective studies using standardized NS definitions are warranted.

## Introduction

Syphilis remains a major global public health concern (1), with increasing incidence reported in many regions (2–5). NS can occur at any stage of infection and may lead to severe neurological complications if not recognized and treated promptly (6–8). However, early identification of patients at risk remains challenging, particularly in the absence of clear neurological symptoms.

Secondary syphilis represents a phase of systemic dissemination of the *Treponema pallidum*, during which central nervous system (CNS) involvement may occur (9). Previous studies have identified several factors associated with NS, including age, sex, serological titres and immune status (10–12). However, data focusing specifically on patients with secondary syphilis—particularly those without HIV infection—remain limited.

In addition, the clinical significance of certain mucocutaneous manifestations, such as oral mucosal patches, which present in 44.1% of secondary syphilis cases (13), has not been well explored in relation to neurological involvement. Identifying clinically accessible markers may help guide decisions regarding Lp in routine practice.

In this study, we aimed to evaluate epidemiological characteristics and factors associated with CSF abnormalities suggestive of NS among HIV-negative patients with secondary syphilis in a large single-center cohort.

## Materials and methods

### Study design and participants

This retrospective study was conducted at sexually transmitted disease clinic at Shanghai Skin Disease Hospital, a tertiary referral center in Shanghai, China. We included consecutive adult patients (≥18 years) diagnosed with secondary syphilis between August 2009 and December 2022.

Secondary syphilis was diagnosed according to national guidelines (14), based on compatible clinical manifestations and serological evidence (reactive non-treponemal test confirmed by treponemal test). Only HIV-negative patients who had not received anti-syphilitic treatment before Lp were eligible. This definition was used as an inclusion criterion for the study population.

All enrolled participants underwent Lp, and completed standard clinical evaluation, including medical history, physical examination, and neurological assessment. Lp was performed in all enrolled participants according to a standardized predefined protocol, and no selection bias was introduced for individuals at increased risk of neurological involvement.

### Data collection and laboratory testing

Clinical and laboratory data were retrospectively extracted from electronic medical records. Variables included demographic characteristics (age, gender, place of residence), clinical features, syphilis history and reinfection, and co-infection with other sexually transmitted infections (STIs).

Serological testing for syphilis was performed using the Toluidine Red Unheated Serum Test (TRUST) and confirmed with the *Treponema pallidum* particle agglutination assay (TPPA). TRUST titres were determined by serial dilution. HIV status was assessed using a chemiluminescence assay.

CSF specimens were analyzed for venereal disease research laboratory (VDRL) reactivity, TPPA, white blood cell (WBC) count, and protein concentration. Additional testing for other STIs, including *Chlamydia trachomatis*, *Neisseria gonorrhoeae*, Mycoplasma species, and human papillomavirus, was performed using polymerase chain reaction (PCR) or standard laboratory methods.

### Case definitions

NS was defined as the presence of syphilis together with either a reactive CSF-VDRL test or CSF abnormalities (elevated protein >0.45 g/L or white blood cell count >5 cells/μL) in the absence of alternative causes (9, 15–17). Given the lack of a universally accepted gold standard, this definition was intended to capture patients with CSF findings suggestive of NS rather than definitive disease.

### Ethics statement

Approval for the study was granted by the Medical Ethics Committee of the Shanghai Skin Disease Hospital (reference number: 2016–011). Written informed consent was obtained from all participants, including those undergoing CSF examinations. The study adhered to the guidelines of the Declaration of Helsinki and all relevant regulations.

### Statistical analysis

Continuous variables were assessed for normality using the Kolmogorov–Smirnov test and are presented as mean (standard deviation, SD) or median (interquartile range, IQR), as appropriate. Comparisons between groups were performed using the t-test or Mann–Whitney U test for continuous variables and the χ^2^ test or Fisher’s exact test for categorical variables.

Univariable logistic regression was used to identify factors associated with NS. Variables with *p* < 0.10 in univariable analysis were included in multivariable logistic regression models to estimate adjusted odds ratios (aORs) and 95% confidence intervals (CIs). Age and serum TRUST titer were dichotomomized at their median values (39 years and 1:64, respectively) to facilitate clinical interpretation and risk stratification in routine practice. CD4^+^T cell count was categorized accroding to the WHO clinical classification (≥500 vs. < 500 cells/μL). This was a restrospective observational study using all consecutive eligible patients; no formal sample size or power calculation was performed before study initiation. The large sample size (*n* = 805) provided sufficient statistical precision for multivariable logistic regression analysis.

Missing data for key variable (age, gender, CD4^+^T cell count, TRUST titer, CSF parameters) were less than 5%. Complete-case analysis using multiple imputation confirmed that missing data did not materially alter the main results.

Pre-specified subgroup analyses were performed to explore potential effect modification by age, gender, serum TRUST titer, history of syphilis, reinfection, presence of other STIs, and CD4^+^ cell count. Interaction was assessed using likelihood ratio tests. All statistical tests were two-sided, with *p* < 0.05 considered statistically significant.

All analyses were conducted using SPSS Statistics version 26.0 (Chicago, IL, United States) and R software version 4.2.1 Graphs were generated using the ggplot2 package version 3.4.4.

## Results

### Study population

Between August 2009 and December 2022, 6,886 individuals with newly diagnosed secondary syphilis were screened. Of these, 1,050 participants who provided informed consent underwent further evaluation. After exclusion of 236 individuals with HIV co-infection and 9 individuals aged <18 years, 805 patients were included in the final analysis.

**Figure 1:**
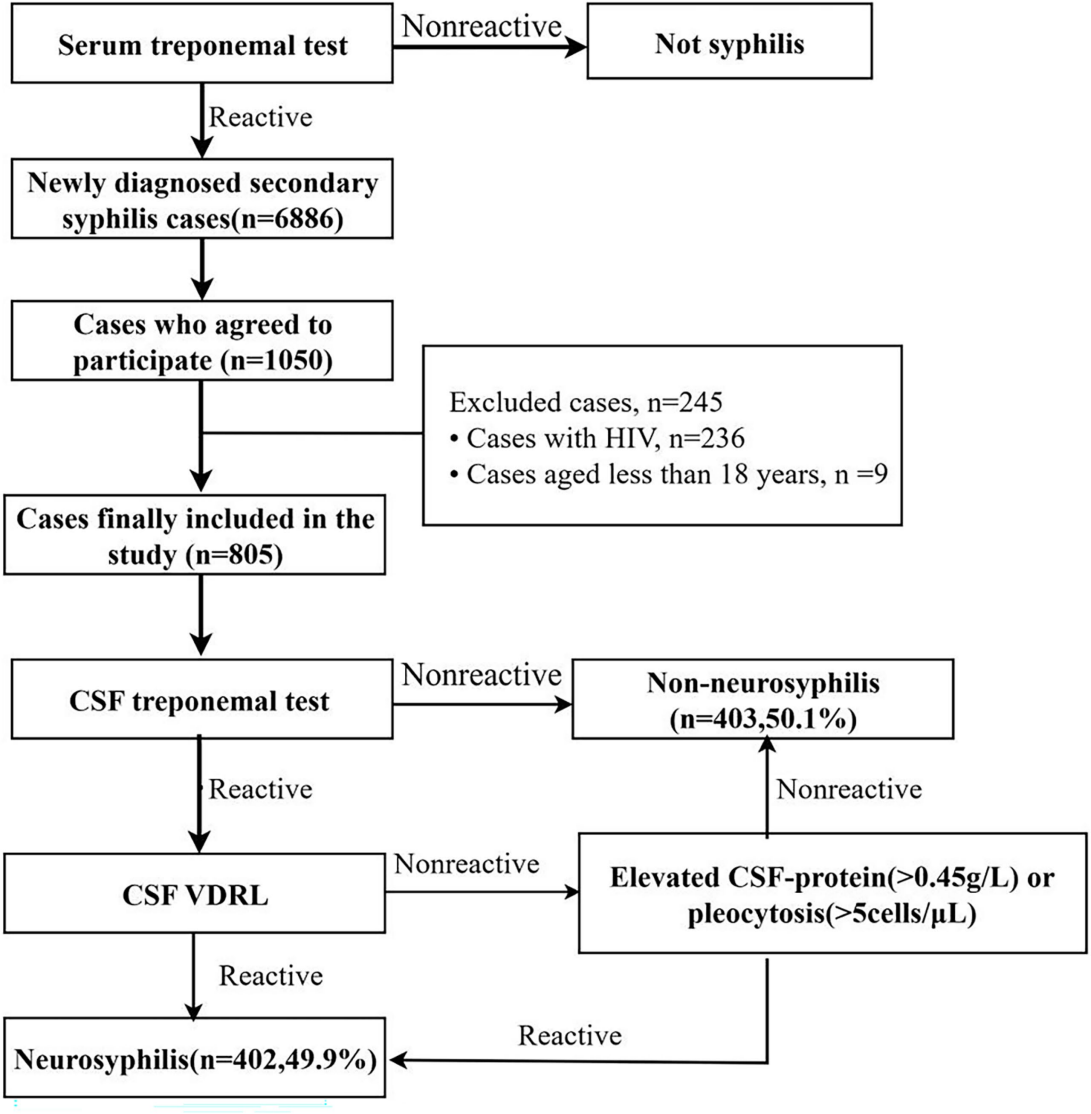
Enrollment flow chart of patients with secondary syphilis. From an initial cohort of 6,886 secondary syphilis cases diagnosed between August 2009 and December 2022, 1,050 patients provided informed consent and underwent lumbar puncture. After excluding 236 individuals with HIV co-infection and 9 individuals aged under 18 years, 805 HIV-negative adult patients were included in the final analysis. Participants were classified into NS group (*n* = 402,49.9%) and non-NS group (*n* = 403,50.1%) based on CSF-VDRL reactivity or CSF abnormalities (CSF protein >0.45 g/L or CSF WBC > 5 cells/μL) in the absence of alternative causes. NS, neurosyphilis; HIV, human immunodeficiency virus; CSF, cerebrospinal fluid; VDRL, Venereal Disease Research Laboratory.

Among these participants, 402 (49.9%) met the study definition of NS based on CSF findings, while 403 patients (50.1%,) did not. The patient selection process is shown in Figure 1.

### Baseline characteristics

The median age of the study participants was 39 years (IQR: 29, 54). Patients who met the study definition of NS were older than those who did not (median 52 vs. 37 years, *p* < 0.001). Overall, 55.2% of participants were male. A higher proportion of males was observed in the NS group compared with the non-neurosyphilis (non-NS) group (64.4% vs. 45.9%, *p* < 0.001). Common mucocutaneous manifestation of secondary syphilis included generalized rash, condyloma latum, and oral mucosal patches. In addition to oral mucosal patches, other atypical cutaneous or mucosal lesions were observed in the subset of patients but were not systematically collected for the primary analysis.

Oral mucosal lesions were more frequently observed in the NS group (18.2%) than in the non-NS group (7.7%) (*p* < 0.001). Syphilis reinfection was also more common in the NS group (26.1% vs. 17.4%, *p* = 0.003). There were no significant differences between groups in history of syphilis or co-infection with other STIs.

The median serum TRUST titer was 1:64 (IQR 1:32–1:128) in the overall population, with a modest but statistically significant difference between groups (*p* = 0.009). CD4^+^T-cell counts were comparable between groups (*p* = 0.147). Detailed characteristics are presented in Table 1.

**Table 1:** Demographic and clinical characteristics of the study population.

Characteristics	Total (*n* = 805) median (IQR)/(N%)^1^	Non-NS group (*n* = 403) median (IQR)/(N%)^1^	NS group (*n* = 402) median (IQR)/(N%)^1^	*P*-value^2^
Age, years	39(29, 54)	37(28, 50.25)	52(38, 60)	<0.001
Gender				<0.001
Female	361(44.8%)	218(54.1%)	143(35.6%)	
Male	444(55.2%)	185(45.9%)	259(64.4%)	
Domicile place				<0.001
Shanghai Province	502(62.4%)	227(56.3%)	275(68.4%)	
Other provinces	303(37.6%)	176(43.7%)	127(31.6%)	
History of syphilis				0.292
No	635 (78.9%)	324 (80.4%)	311 (77.4%)	
Yes	170 (21.1%)	79 (19.6%)	91 (22.6%)	
Syphilis reinfection				0.003
No	630 (78.3%)	333 (82.6%)	297 (73.9%)	
Yes	175 (21.7%)	70 (17.4%)	105 (26.1%)	
Serum TRUST titer	1:64(1:32, 1:128)	1:64 (1:32, 1:64)	1:64(1:32, 1:64)	0.009
Oral mucous patches				<0.001
No	701 (87.1%)	372 (92.3%)	329 (81.8%)	
Yes	104 (12.9%)	31 (7.7%)	73 (18.2%)	
Other STIs				0.533
No	537 (66.7%)	273(67.7%)	264 (65.7%)	
Yes	268 (33.3%)	130 (32.3%)	138 (34.3%)	
CD4^+^T cell count (cells/μL)	643(516,643)	643(530,643)	643(487,678.5)	0.147

^1^Median [IQR]; n (%). ^2^Wilcoxon rank sum test; Pearson’s Chi-squared test; Fisher’s Exact Test for Count Data with simulated *p*-value (based on 2000 replicates). Other STIs, *Neisseria gonorrhoeae*, chlamydia, mycoplasma, herpes (HSV 2) and human papilloma virus (HPV 6, 11, 16, and 18). NS, Neurosyphilis; TRUST, Toluidine Red Unheated Serum Test.

### Factors associated with CSF abnormalities suggestive of NS

In univariable logistic regression analysis, age over 39 > years, male gender, serum TRUST titer ≥ 1:64, syphilis reinfection, presence of oral mucous patches, and a CD4^+^T cell count < 500 cells/μL were associated with an increased odds of meeting the study definition of NS.

In multivariable analysis, age > 39 years (adjusted OR [aOR] 2.72, 95% CI 2.01–3.68). male gender (aOR 2.14, 95% CI 1.58–2.90), oral mucosal lesions (aOR 2.21, 95% CI 1.38–3.55), and a CD4^+^T cell count < 500 cells/μL (aOR 1.46, 95% CI 1.02–2.10) remained independently associated with NS. Serum TRUST titer and syphilis reinfection were not statistically significant after adjustment (Table 2).

**Table 2:** Predictors of neurosyphilis according to univariable and multivariate regression.

Characteristics	Total (N)	OR (95%CI) univariate analysis	*P*-value univariate analysis	OR (95%CI) Multivariate analysis	*P-*value Multivariate analysis
Age, years	805				
≤39	412	Reference		Reference	
>39	393	2.99 (2.245–3.984)	<0.001	2.72(2.013–3.682)	<0.001
Gender					
Female	361	Reference		Reference	
Male	444	2.13 (1.608–2.832)	<0.001	2.14 (1.582–2.900)	<0.001
Serum TRUST titer	805				
<1:64	256	Reference		Reference	
≥1:64	549	1.34(0.997–1.809)	0.052	1.18(0.854–1.617)	0.322
History of syphilis					
No	635	Reference			
Yes	170	1.20 (0.855–1.685)	0.292		
Syphilis reinfection					
No	630	Reference		Reference	
Yes	175	1.69(1.196–2.364)	0.003	1.28 (0.885–1.855)	0.19
Oral mucous patches					
No	701	Reference		Reference	
Yes	104	2.66 (1.705–4.157)	<0.001	2.21 (1.377–3.545)	0.001
Other STIs					
No	537	Reference			
Yes	268	1.10(0.819–1.472)	0.533		
CD4^+^T cell count (cells/μL)					
≥500	625	Reference		Reference	
<500	180	1.64(1.171–2.295)	0.004	1.46(1.018–2.095)	0.04

In the analysis, age and serum TRUST titer were dichotomized at their median values, and groups were defined accordingly. CD4 cell counts were grouped according to the WHO classification.

### Subgroup analyses

**Figure 2:**
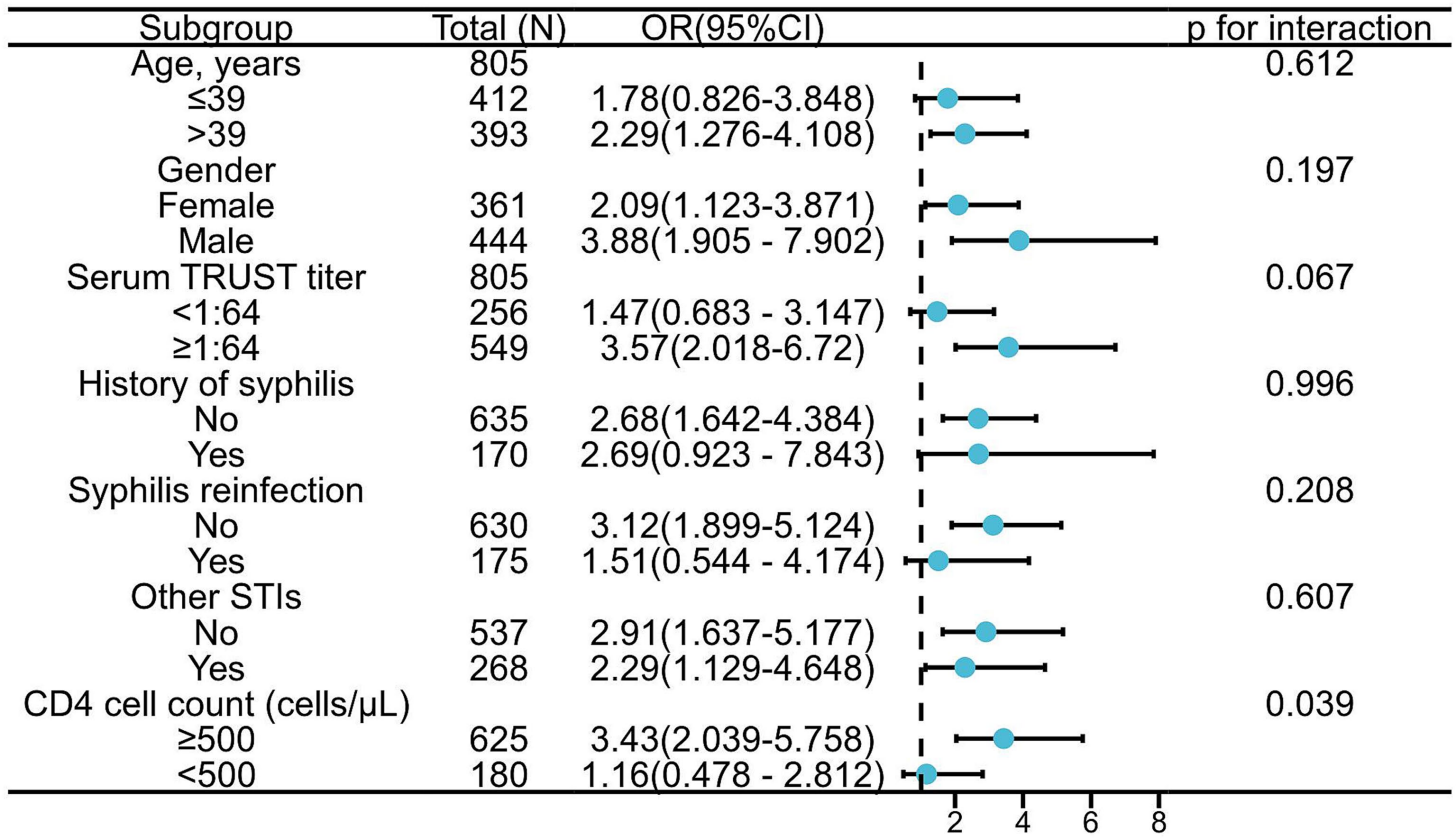
Association between neurosyphilis and oral mucous patches in subgroup analyses Forest plot showing the odds ratios (ORs) and 95% confidence intervals (CIs) for the association between oral mucous patches and NS across prespecified subgroups. The vertical dashed line represents an OR of 1 (null effect). Age and serum TRUST titer were dichotomized at median values. CD4^+^T-cell count was grouped according to WHO classification. A significant interaction was observed for CD4^+^T-cell count (p for interaction = 0.039), indicating that the association differed by immune status. A near-significant interaction was found for serum TRUST titer (p for interaction = 0.067). No significant interactions were detected for age, gender, syphilis history, syphilis reinfection, or other STIs (all *p* > 0.05). OR, odds ratio; CI, confidence interval; NS, neurosyphilis; TRUST, Toluidine Red Unheated Serum Test; STIs, sexually transmitted infections.

Subgroup analyses were conducted to explore whether the association between oral mucosal lesions and NS differed across clinical and demographic strata (Figure 2). The presence of oral mucosal lesions was consistently associated with higher odds of NS across most subgroups.

The association appeared more pronounced among older individuals (>39 years) and males. A statistically significant interaction was observed for CD4^+^ T-cell count (p for interaction = 0.039), suggesting that the association between oral mucosal lesions and NS may vary according to immune status. However, given the exploratory nature of these analyses, the findings should be interpreted with caution.

## Discussion

In this large retrospective study of HIV-negative patients with secondary syphilis, we identified several factors associated with CSF abnormalities suggestive of NS, including older age, male sex, oral mucosal lesions, and lower CD4^+^T cell counts. These findings may assist in identifying patients who could benefit from further neurological evaluation in clinical practice.

Our findings are consistent with previous studies that have reported associations between NS and demographic and immunological factors, particularly older age and male sex (18). These results underscore the importance of including age-stratified immunological variables in NS risk models. Prior research has also highlighted the role of serological titres and host immune status in influencing CNS involvement (7, 12, 19, 20). However, many earlier studies have included heterogeneous populations across different stages of syphilis or have focused on individuals with HIV co-infection (21–24). By restricting our analysis to HIV-negative patients with secondary syphilis, the present study provides a more focused assessment within a clinically important disease stage.

The association between oral mucosal lesions and CSF abnormalities is of particular interest. Rather than suggesting a direct causal relationship, oral mucosal involvement may reflect a higher treponemal burden or systemic infection or a more pronounced inflammatory response (25–27). As such, these lesions could serve as a readily identifiable clinical marker to support risk stratification. Their presence, especially when combined with other factors such as age or sex, may help clinicians identify patients who warrant further neurological assessment. We acknowledge that secondary syphilis may present with a spectrum of atypical cutaneous and mucosal manifestations, which have been reported to account for up to 25% of cases (28). In the present study, we focused specifically on oral mucosal patches because they are common, readily identifiable, and understudied in relation to central nervous system involvement. Other atypical manifestations were not included in the primary analysis and warrant further investigation in future studies.

We also observed a relationship between lower CD4^+^T cell counts and NS in HIV-negative patients with secondary syphilis. This finding suggests that variation in host immune function, even outside the context of HIV infection, may influence susceptibility to CNS involvement (29, 30). While the underlying mechanisms remain unclear, this observation supports the broader concept that host immune status plays a role in the pathogenesis of NS and merits further investigation.

From a clinical perspective, these findings may have implications for decision-making regarding Lp in patients with secondary syphilis. Given that neurological symptoms may be absent or non-specific in early disease, identifying clinical features associated with CSF abnormalities could help guide more targeted use of invasive diagnostic procedures. In particular, the presence of oral mucosal lesions in combination with older age or male sex may prompt consideration of CSF evaluation, even in the absence of overt neurological manifestations.

Several limitations should be considered when interpreting these findings. First, the retrospective design and inclusion of only patients who underwent Lp based on clinical judgment may have introduced selection bias, potentially overrepresenting individuals at higher risk of neurological involvement. As a result, the observed prevalence of NS in this cohort may not be generalizable to the broader population of patients with secondary syphilis. Second, the NS definition used in this study was broad, based on CSF-VDRL reactivity or non-specific CSF abnormalities in the absence of alternative causes. This definition was intended to identify CSF abnormalities suggestive of NS rather than definitive disease, which may have led to overestimation of NS prevalence. Third, the single-center design may limit generalizability to other settings and populations. Fourth, our study focused on oral mucosal patches and did not systematically analyze the full spectrum of atypical secondary syphilis manifestations and their association with CSF abnormalities. Finally, the absence of longitudinal follow-up precludes assessment of clinical outcomes and the prognostic value of the identified factors.

In conclusion, among HIV-negative patients with secondary syphilis undergoing Lp, several demographic, clinical, and immunological factors were associated with CSF abnormalities suggestive of NS. These findings may support more targeted neurological evaluation in selected patients. Further prospective, multi-center studies using standardized diagnostic criteria are needed to validate these observations and clarify their clinical significance.

## Data Availability

The original contributions presented in the study are included in the article/supplementary material, further inquiries can be directed to the corresponding author.
